# Fatal Nongroupable *Neisseria meningitidis* Disease in Vaccinated Patient Receiving Eculizumab

**DOI:** 10.3201/eid2408.180228

**Published:** 2018-08

**Authors:** Deirdre Nolfi-Donegan, Monica Konar, Vianca Vianzon, Jessica MacNeil, James Cooper, Perrianne Lurie, Judi Sedivy, Xin Wang, Dan M. Granoff, Lucy McNamara

**Affiliations:** Children’s Hospital of Pittsburgh, Pittsburgh Pennsylvania, USA (D. Nolfi-Donegan, J. Cooper);; The University of California, San Francisco Benioff Children's Hospital Oakland, Oakland, California, USA (M. Konar, V. Vianzon, D.M. Granoff);; Centers for Disease Control and Prevention, Atlanta, Georgia, USA (J. MacNeil, X. Wang, L. McNamara);; Pennsylvania Department of Health, Harrisburg, Pennsylvania, USA (P. Lurie, J. Sedivy)

**Keywords:** paroxysmal nocturnal hemoglobinuria, MenB-4C, 4CMenB, Bexsero, Soliris, eculizumab, meningococcemia, bacteria, vaccines, vaccinated, immunization, meningitis/encephalitis, meningococci

## Abstract

Patients receiving eculizumab have an increased risk for meningococcal disease, but most reported cases are attributable to encapsulated meningococcal strains. We describe a case in which a nongroupable meningococcal strain, which rarely causes disease in healthy persons, caused fatal disease in an eculizumab recipient despite meningococcal vaccination.

Eculizumab (Soliris; Alexion Pharmaceuticals, New Haven, CT, USA) is currently the only disease-modifying agent approved for patients with paroxysmal nocturnal hemoglobinuria (PNH) and atypical hemolytic uremic syndrome. Eculizumab decreases complement-induced hemolysis by preventing cleavage of C5 into C5a and C5b; however, this activity also prevents formation of the membrane attack complex that is essential for meningococcal serum bactericidal activity (*1*).

The Food and Drug Administration–approved eculizumab prescribing information (*2*) includes a warning for increased risk for meningococcal disease and requires a Risk Evaluation and Mitigation Strategy (*3*) to ensure patient and prescriber awareness of the meningococcal disease risk and need for meningococcal vaccination. However, meningococcal disease occurs in eculizumab recipients despite appropriate vaccination (*4*–*6*). Most reported cases have been caused by encapsulated isolates. We describe a fully vaccinated patient with PNH who received 2 doses of eculizumab and died shortly thereafter from overwhelming *Neisseria meningitidis* disease caused by nongroupable meningococci, which rarely cause disease in human hosts.

## Case Report

In March 2016, a 16-year-old previously healthy girl was brought to a children’s hospital with abdominal pain, pancytopenia, and laboratory evidence of hemolysis. After extensive work-up, she received a diagnosis of PNH on the basis of peripheral blood flow cytometry. A bone marrow biopsy demonstrated cellularity ≈40%–50% and complete myeloid and erythroid maturation without evidence of dysplasia, aplasia, or an aberrant cell population. In anticipation of possible future eculizumab immunotherapy, she received a booster vaccine targeting *N. meningitidis* serogroups A, C, Y, and W-135 (Menactra; Sanofi Pasteur, Inc., Swiftwater, PA, USA) and a 2-dose series of vaccine targeting *N. meningitidis* serogroup B (MenB-4C) (Bexsero; GlaxoSmithKline, Bellaria Rosia, Sovicille, Italy).

During the first 4 months after diagnosis, the patient exhibited mild pancytopenia and compensated hemolysis but remained transfusion-independent. However, 6 months after PNH diagnosis, she began eculizumab treatment because of worsening symptoms. 

Twenty-two hours after her second dose of eculizumab, the patient reported to a local emergency department with generalized body pain, headache, and emesis. She was afebrile, and her physical examination was unremarkable except for tachycardia of 124 beats per minute. Her leukocyte count was 8.9 × 10^9^ cells/L (within reference limits); absolute neutrophil count was 8.46 × 10^9^/L (upper limit 8.00 × 10^9^ cells/L). Her symptoms were attributed to side effects from eculizumab and resolved after treatment with prochlorperazine, ketorolac, diphenhydramine, and intravenous fluids. She was discharged to home after 2 hours of observation.

Approximately 12 hours later, the patient reported weakness and purpura developed. She experienced cardiac arrest in transit to another emergency department, and resuscitative efforts were unsuccessful. Autopsy revealed hemorrhagic necrosis of the adrenal glands and focal hemorrhagic skin purpura, consistent with Waterhouse-Friderichsen syndrome.

By whole-genome sequencing, the meningococcal strain isolated from the meninges was found to be sequence type (ST) 2578 (clonal complex ST-41/44) and nongroupable with a capsule null locus, *cnl*. The inferred amino acid sequences of Factor H binding protein (FHbp) (peptide ID 100) and *Neisseria* heparin binding antigen (NHba) (peptide ID 2) were 97% and 100% identical to the respective antigens in the MenB-4C vaccine the patient had received, and both antigens were expressed on the surface of live bacteria based on flow cytometry. The remaining MenB-4C antigens were either absent (NadA) or mismatched (strain PorA P1,17,9).

Using 15% IgG-depleted human serum as a complement source, we found the strain was susceptible (titer >40) to bactericidal activity of mouse antiserum to recombinant FHbp ID 1, the subfamily B subvariant in the MenB-4C vaccine but not to mouse antiserum to NadA (not expressed by the strain) or to FHbp ID 22 (a subfamily A subvariant not in the vaccine) (titers <10). Mouse anti-NHba antiserum had a titer of <10. The relative resistance to anti-NHba was similar to previous findings that some NHba-expressing serogroup B meningococcal strains resist MenB-4C vaccine–elicited anti-NHba serum bactericidal activity (SBA) in humans (*7*).

**Figure 1:**
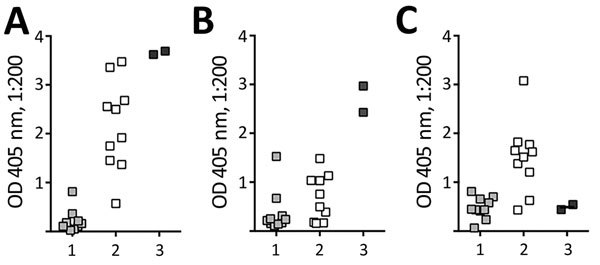
Serum IgG reactivity to 3 recombinant antigens in *Neisseria meningitidis* serogroup B meningococcal vaccine (MenB-4C) (Bexsero; GlaxoSmithKline, Bellaria Rosia, Sovicille, Italy), determined by ELISA. A postmortem serum sample (*3*) from a 16-year-old girl with paroxysmal nocturnal hemoglobinuria who died of meningococcal disease after treatment with eculizumab. Reactivity was measured in parallel with stored serum from 10 unvaccinated college students (*1*) and 10 vaccinated college students (*2*) 7 months after vaccination with MenB-4C (*8*). For the comparison specimens, each data point represents reactivity of an individual person. A) Factor H binding protein. B) *Neisseria* heparin binding antigen. C) NadA, MenB-4C antigens absent. OD, optical density. Data points for (*3*) indicate results of replicate assays. OD, optical density.

A postmortem serum sample had high IgG reactivity against FHbp and NHba, both expressed by the infecting strain, and low reactivity to NadA (Figure 1). The high IgG reactivity specific for the 2 vaccine antigens expressed by the strain, but not for the vaccine antigen absent in the strain, suggests these antibodies may represent an IgG memory response elicited by infection. Given the short duration of symptoms, the likely stimulus for the memory antibody response would be asymptomatic nasopharyngeal colonization before disease onset.

**Figure 2:**
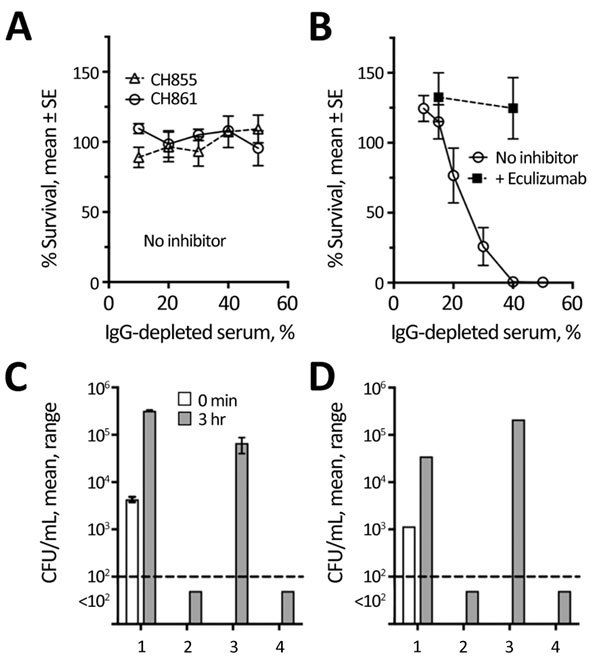
Effect of eculizumab on serum bactericidal activity and killing of *Neisseria meningitidis* by anticoagulated human blood. A) Complement-mediated bactericidal activity of an IgG-depleted human serum pool from 3 unvaccinated adult donors measured against encapsulated serogroup B strains CH855 and CH861 (data from 2–4 replicate assays for each strain). B) Bactericidal activity of pool tested in (panel A) measured against the nongroupable (NG) case isolate from a 16-year-old girl with paroxysmal nocturnal hemoglobinuria who died of meningococcal disease after treatment with eculizumab (data from 5 replicate assays). The addition of 50 μg/mL of eculizumab blocked bacterial killing of the NG case-isolate (data from 3 replicates). C, D) Killing of the NG case-isolate by anticoagulated human blood from 2 healthy adults, 1 previously vaccinated with 2 doses of *Neisseria meningitidis* serogroup B meningococcal vaccine (MenB-4C) (Bexsero; GlaxoSmithKline, Bellaria Rosia, Sovicille, Italy) with the last dose 14 months earlier (C), the other unvaccinated (D). The addition of 50 μg/mL of eculizumab to the blood from both donors blocked killing of the bacteria by the blood. The addition of a mouse anti-C7 monoclonal antibody, which blocked serum bactericidal activity (data not shown), did not inhibit whole blood killing. Similar results were obtained with blood from a third adult who had been vaccinated with MenB-4C 9 months earlier (data not shown). 1, negative control (plasma-heated at 56°C for 30 min to inactivate complement activity); 2, no inhibitor; 3, eculizumab; 4, anti-C7 (24 whole blood assay).

We also measured bactericidal activity against the patient isolate using pooled IgG-depleted serum from 3 healthy unvaccinated adults, performed as previously described (*9*). Whereas 2 invasive serogroup B encapsulated strains survived in the IgG-depleted serum pool (Figure 2, panel A), the nongroupable case-strain was killed (Figure 2, panel B), which could be from complement alone or complement activated by naturally acquired IgM. However, 50 µg/mL of eculizumab (a concentration less than or equal to trough serum levels in treated patients [*10*]) completely blocked killing of the case-isolate (Figure 2, panel B).

Finally, we tested eculizumab’s effect on whole blood killing of the unencapsulated case-isolate in an assay that measures a combination of SBA and opsonophagoctyic (OPA) activity (*11*,*12*). Cultures were sterile after 1–3 hours incubation in anticoagulated whole blood from MenB-4C–vaccinated or –unvaccinated adults (Figure 2, panels C, D), but 50 µg/mL eculizumab completely blocked bacterial killing. In contrast, the addition of a mouse monoclonal antibody to C7 (required for SBA but not OPA) did not prevent killing. These findings extend prior studies of whole blood killing of encapsulated serogroup B and C strains, which demonstrated that blocking C5 cleavage inhibited both SBA and OPA (*11*,*12*) killing of these strains, to a nonencapsulated meningococcal strain.

## Conclusions

Collectively, these data indicate that eculizumab-treated patients remain profoundly susceptible to meningococcal disease, including from nongroupable meningococcal strains. Neither prior vaccination with MenB-4C, which matched 2 antigens present in the strain, nor the high serum antibody levels to FHbp and NHba in the patient prevented rapidly fatal disease in the presence of eculizumab. Similarly, eculizumab blocked killing of the nongroupable isolate by whole blood from healthy unvaccinated and vaccinated adults.

This case, along with several additional cases of meningococcal disease caused by nongroupable strains, was recently reported in eculizumab recipients in the United States (*13*). In otherwise healthy hosts, virtually all invasive meningococcal disease is caused by encapsulated strains (*14*). In contrast, unencapsulated strains are commonly associated with asymptomatic nasopharyngeal carriage. The sequence type of the isolate from this case, ST-2578, is rarely found in the PubMLST *Neisseria* database but previously has been observed primarily among isolates from asymptomatic carriers (*15*). Furthermore, the strain was killed by complement in human serum that had been depleted of IgG and by whole blood from an unvaccinated adult, features of commensal meningococcal strains that lack capsules.

In the United States, there is no official guidance on the use of antimicrobial chemoprophylaxis in eculizumab recipients. However, France and the United Kingdom recommend chemoprophylaxis for the duration of eculizumab therapy (references *16,17* in Technical Appendix), consistent with recommendations in recent case reports (*1*,*13*; reference *18* in Technical Appendix). Most chemoprophylactic regimens for eculizumab-treated patients use penicillin or erythromycin (*1*; reference *18 *in Technical Appendix). Although the strain isolated from the patient reported was susceptible to penicillin, meningococcal disease has been reported in eculizumab patients receiving penicillin chemoprophylaxis caused by strains with penicillin resistance or intermediate sensitivity (*5*; reference *19* in Technical Appendix). These breakthrough cases underscore the need for healthcare providers and patients to have a high index of suspicion for meningococcal disease, leading to quick recognition and consideration of early empiric treatment, regardless of the patient’s vaccination status or chemoprophylactic regimen.

Technical Appendix Additional references.
