# Supplementary material for: Fatal Nongroupable Neisseria meningitidis Disease in Vaccinated Patient Receiving Eculizumab
Source: Emerg Infect Dis. 2018 Aug;24(8):1561–4. doi: 10.3201/eid2408.180228 (PMC6056112; doi:10.3201/eid2408.180228)
Supplement: Technical Appendix — Additional references. [file 18-0228-Techapp-s1.pdf]

Article DOI: https://doi.org/10.3201/eid2408.180228

Nongroupable *Neisseria meningitidis* Disease in Vaccinated Patient Receiving Eculizumab

Technical Appendix

Additional References

16. Haut Conseil de la Santé Publique. Personnes traitées par Soliris®: actualisation des recommandations de vaccination et d'antibioprophylaxie [cited 2017 Jul 23]. http://www.hcsp.fr/Explore.cgi/avisrapportsdomaine?clefr = 447

17. PNH National Service Leeds. Meningococcal infection and eculizumab [cited 2016 Dec 2]. http://www.pnhleeds.co.uk/professionals/meningococcal-infection-and-eculizumab/

18. Benamu E, Montoya JG. Infections associated with the use of eculizumab: recommendations for prevention and prophylaxis. Curr Opin Infect Dis. 2016;29:319–29. PubMed http://dx.doi.org/10.1097/QCO.0000000000000279

19. Parikh SR, Lucidarme J, Bingham C, Warwicker P, Goodship T, Borrow R, et al. Meningococcal B vaccine failure with a penicillin-resistant strain in a young adult on long-term eculizumab. Pediatrics. 2017;140:e20162452. PubMed http://dx.doi.org/10.1542/peds.2016-2452
